# Oral administration of hepcidin and chitosan benefits growth, immunity, and gut microbiota in grass carp (*Ctenopharyngodon idella*)

**DOI:** 10.3389/fimmu.2022.1075128

**Published:** 2022-12-15

**Authors:** Jiancheng Zhou, Mengzhen Feng, Weixiang Zhang, Rui Kuang, Qi Zou, Jianguo Su, Gailing Yuan

**Affiliations:** ^1^ Department of Aquatic Animal Medicine, College of Fisheries, Huazhong Agricultural University, Wuhan, Hubei, China; ^2^ Wuhan DaBeiNong (DBN) Aquaculture Technology Co. LTD, Wuhan, Hubei, China

**Keywords:** hepcidin, chitosan, *Ctenopharyngodon idella*, growth, immunity, gut microbiota

## Abstract

Intensive high-density culture patterns are causing an increasing number of bacterial diseases in fish. Hepcidin links iron metabolism with innate immunity in the process of resisting bacterial infection. In this study, the antibacterial effect of the combination of hepcidin (Cihep) and chitosan (CS) against *Flavobacterium columnare* was investigated. The dosing regimen was also optimized by adopting a feeding schedule of every three days and every seven days. After 56 days of feeding experiment, grass carp growth, immunity, and gut microbiota were tested. *In vitro* experiments, Cihep and CS can regulate iron metabolism and antibacterial activity, and that the combination of Cihep and CS had the best protective effect. *In vivo* experiments, Cihep and CS can improve the growth index of grass carp. After challenge with *Flavobacterium columnare*, the highest survival rate was observed in the Cihep+CS-3d group. By serum biochemical indicators assay and Prussian blue staining, Cihep and CS can increase iron accumulation and decrease serum iron levels. The contents of lysozyme and superoxide dismutase in Cihep+CS-3d group increased significantly. Meanwhile, Cihep and CS can significantly reduce the pathological damage of gill tissue. The 16S rRNA sequencing results showed that Cihep and CS can significantly increase the abundance and diversity of grass carp gut microbiota. These results indicated that the protective effect of consecutive 3-day feeding followed by a 3-day interval was better than that of consecutive 7-day feeding followed by a 7-day interval, and that the protective effect of Cihep in combination with chitosan was better than that of Cihep alone. Our findings optimize the feeding pattern for better oral administration of Cihep in aquaculture.

## Introduction

In China, freshwater fish farming is an important industry, accounting for 70% of the world’s aquatic products (1). Grass carp (*Ctenopharyngodon idella*), a herbivorous freshwater fish, is one of the four domestic fishes in China, and it ranks the first in the production yield among freshwater farmed fishes (2). Data from the 2020 Fisheries Yearbook show that grass carp production yield reached 5.533 million tons in 2019, accounting for 18.36% of freshwater aquaculture production. However, the frequent occurrence of diseases has become an important limiting factor for the sustainable and healthy development of grass carp farming industry (3). Intensive high-density culture patterns are causing an increasing number of bacterial diseases in fish. *Flavobacterium columnare* (*F. columnare*) is a common pathogen of freshwater cultured fish. *F. columnare* can cause bacterial gill rot disease. In recent years, there have been outbreaks of the disease due to intensive farming (4, 5).

Antibiotics are widely used for controlling diseases. However, the overuse of antibiotics can lead to excessive accumulation of antibiotics in the water environment as well as in aquatic organisms, which in turn can be harmful to human health (6). In addition, overuse of antibiotics may also lead to bacterial resistance, thereby reducing the immunity of aquatic animals (7, 8). Therefore, avoiding the excessive use of antibiotics in the aquaculture industry has become one of the major challenges facing human health and the aquaculture sector (9).

Antimicrobial peptides (AMPs) are seen as the most promising alternative to antibiotics (10, 11). Hepcidin is a cysteine-rich peptide hormone synthesized mainly by hepatocytes (12–14). Krause and Park et al. successively extracted hepcidin from human plasma and urine in 2001 (15, 16). Hepcidin can combine with ferroportin and cause the ubiquitination of ferroportin, thus reducing the serum iron content of the body (17). Hepcidin can promote the expression of certain immune-related genes, thereby enhancing the defense function of fish against pathogens (18–20). Hepcidin can also limit the uptake and utilization of iron by pathogens by decreasing the iron level in organism (21, 22). Our previous studies have demonstrated that hepcidin can reduce mortality caused by bacterial infections in fish by regulating iron metabolism and immune-related gene expression (23–26). Therefore, hepcidin-regulated iron removal is an important method to control bacterial growth and improve survival.

Chitosan is an important immune enhancer and is the only alkaline polysaccharide present in nature, mainly found in the shells of aquatic animals such as shrimps, crabs, and shellfish (27). In recent years, chitosan has been widely used in aquaculture industry for its advantages of promoting aquatic animal growth and improving immunity and disease resistance of aquatic animals (28–30). Furthermore, dietary chitosan has no toxic effect on its biodegradability and biocompatibility (31). Studies have shown that chitosan can increase the microbial diversity of the fish gut and participate in the immune regulatory system of the body by regulating the ratio of beneficial and pathogenic microorganisms (32). More specifically, beneficial bacteria secrete digestive enzymes and nutrients to promote intestinal absorption, which in turn improves fish growth rate and feed efficiency (32). In addition, adding chitosan to feed can improve the antioxidant properties of aquatic organisms (33).

Previous studies in our laboratory have shown that hepcidin have dual functions as iron-regulating and antimicrobial activity. Chitosan, as a widely used immune polysaccharide in aquaculture, also plays an important role in improving the immunity of fish. In view of this, we explored the effectiveness of different hepcidin feeding methods or its combination with chitosan on growth, immunity, and gut microbiota of grass carp. Meanwhile, we analyzed the iron-regulation function of hepcidin and the specific role in the immune system of grass carp infected with *F. columnare*. This study aims to develop new green and safe antibiotic alternatives so as to provide theoretical basis for controlling diseases of cultured fish.

## Materials and methods

### The grass carp management

Grass carp (150.53 ± 1.80 g) obtained from the Da Bei Nong Recirculating Water Farming Base (Wuhan, China) were maintained and acclimated in a recirculating freshwater system with daily feeding. Before experiment, the grouped grass carp were temporarily raised for 7 days for acclimatization. During the experiments, feeding was scheduled at 8: 00 am and 16:00 pm twice a day, and the feeding rate was 2%. During the experiment, the water temperature was maintained at 25-28 °C, the pH was 7.0-7.5, the dissolved oxygen was greater than 5 mg/L, the concentration of ammonia was less than 0.2 mg/L, and nitrite concentration was less than 0.01 mg/L. Treatment methods of experimental animals involved in this experiment comply with the principles of experimental animal welfare and have been approved by the Animal Committee of Huazhong Agricultural University. The ethics number is HZAUFI-2021-0012.

### Experimental design

Fish cultivation experiment lasted 56 days. At the start of the experiment, 450 grass carp were randomly distributed into 15 fiberglass tanks (300 L of water) with 30 grass carp per tank. The grouping was as follows: the control group (Control) was fed with basal diet on daily basis; the Cihep group (Cihep-3d) was fed with Cihep for three days on end followed by three-day interval; the Cihep group (Cihep-7d) was fed with Cihep for seven days on end followed by seven-day interval; the Cihep+chitosan group (Cihep+CS-3d) was fed with Cihep and chitosan for three days on end followed by three-day interval; and the Cihep+ chitosan group (Cihep+CS-7d) was fed with Cihep and chitosan for seven days on end followed by seven-day interval. The feed formula of each group is shown in **Table 1**.

**Table 1:** Experimental feed formula.

IngredientsContent (g/1000 g feed)	Control	Cihep-7d	Cihep-3d	Cihep+CS-3d	Cihep+CS-7d
Soybean meal	360.01	360.01	360.01	359.98	359.98
Rapeseed meal	260.00	260.00	260.00	260.00	260.00
Vegetable cake	115.00	115.00	115.00	115.00	115.00
Wheat	170.00	170.00	170.00	170.00	170.00
Soybean oil	50.00	50.00	50.00	50.00	50.00
Vitamin premix	12.00	12.00	12.00	12.00	12.00
Mineral premix	8.00	8.00	8.00	8.00	8.00
Ca(H_2_PO_4_)	25.00	25.00	25.00	25.00	25.00
Hepcidin	0	0.09	0.09	0.09	0.09
Chitosan	0	0	0	0.03	0.03

### The prokaryotic expression of grass carp hepcidin

DNAs encoding the open reading frame (ORF) of the *C. idella* hepcidin gene (Cihep; GenBank: JQ246442.1) were inserted into the *E. coli* expression vector pGEX-KG (Novagen). The confirmed recombinant expression construct pGEXKG-Cihep and the expression construct pGEX-KG were transformed into competent *E. coli* BL21 (DE3) cells (Novagen). Then, the transformed cells were inoculated into 600 mL LB medium (LB, L3027, Sigma, Shanghai, China) in the fermenter, incubated at 37°C at 150 rpm for 3 h, and cultured to an OD600 of 2.0–5.0. It was then cooled to 25°C, before a final concentration of 1 mM IPTG was added to induce expression for 5 h. After induction, the bacterial solution was centrifuged at 20,000 r/min, collected, weighed, and stored at −80°C. The cell pellet was then resuspended in PBS buffer at 25°C. The cells were subsequently broken by passing them through a French pressure cell press at 1.0 × 10^3^ bar for 5 min. After centrifugation at 12,000g for 1 h at 4°C, the recombinant Cihep was obtained. Next, recombinant protein was prepared as freeze-drying powder and detected using SDS-PAGE electrophoresis. Finally, stored at -20 °C for the subsequent use.

### Western blot analysis

Western blot analysis was used to confirm the existence and molecular weights of the obtained Cihep protein. The recombinant Cihep protein was separated on a 12% SDS-PAGE gel, transferred to nitrocellulose filter membrane at 9 V for 30 min. The membrane was blocked with 5% skimmed milk at room temperature for 1 h, incubated with a 1:4000 dilution of a anti-GST tag mouse monoclonal antibody for 1 h, washed with 1 × TBS + 0.1% Tween and incubated with a 1:5000 dilution of HRP Goat Anti-Mouse secondary antibody (1:5000; 1 mg/mL) (ab6789, Abcam) for 1 h.

### Cell treatment and sample preparation

Grass carp liver cells (L8824) were cultured with M199 medium (Gibco, Beijing, China) in an incubator at 28°C. L8824 cells were directly stimulated by the addition of GST-Cihep fusion protein or CS. The stimulated cells were continued at 28°C. After 24 hours, the medium was decanted and the cells were collected. Cellular was extracted using the Trizol (Aidlab, Beijing, China) method for detection of hepcidin expression. Labile iron Pool (LIP) levels were assessed with reference to the methods covered in previous articles from our laboratory (25).

We assessed the preventive effect of Cihep and CS by culturing L8824 cells with *F. columnare*. Firstly, L8824 cells were cultured in 24-well plates (BeyoGold, Shanghai, China). After 24 hours, cells were continued to be cultured with M199 containing PBS (10,100,147, Gibco, Beijing, China), Cihep, CS, and Cihep + CS. After 3 hours, bacterial suspension (multiplicity of infection MOI=50) was added to each well and the culture was continued for 24 hours. Finally, the cells were fixed with 4% paraformaldehyde solution, and then stained with crystal violet solution for 25 minutes. Finally, the cells were washed and drained. The preventive effect of Cihep and CS was evaluated using crystal violet staining.

### Challenge experiments

*F. columnare* for the challenge experiment was provided by Professor Xie Haixia. The 80 μL of cryopreserved *F. columnare* was inoculated onto a Shieh Agar plate at 26 °C for 48 h to form single colony. The selected single colony was inoculated into the selective medium and incubated at 26 °C for 48 hours. According to the pre-experiment results, the median lethal concentration was 3 ×10^9^ CFU/mL. During the formal challenge, 25 fish were taken from each group for the challenge experiment. Each fish was injected with 3 × 10^9^ CFU/mL of *F. columnare* (500 μL), and the cumulative death curve was obtained in the following 7 days.

### Sample collection and biological analyses

At the end of the 56-day feeding experiment (Pre-challenge), and at 7 d post-challenge, 5 fish were randomly selected from each cylinder after 24 h fasting and anesthetized. Body weight and length were measured to calculate growth index, and blood was collected from the tail vein for analysis of serum biochemical indexes. The fish body was dissected with aseptic dissecting scissors, and the contents of foregut were collected for the analysis of gut microbiota. Hepatopancreas, spleen, and intestines were used for the expression analysis of immune-related genes and iron-related genes by qPCR. The visceral mass and hepatopancreas of grass carp were weighed, and the visceral body ratio and hepatosomatic index were calculated. The hepatopancreas and gill were fixed in 4% paraformaldehyde for histopathological analysis, and the remaining fish were used for challenge experiment.

### Tissue staining

Parts of gill and hepatopancreas tissue were dissected using sterile dissection and immediately fixed with 10% paraformaldehyde. HE staining of gill tissues and Prussian blue staining of hepatopancreas were performed by Borf Biotechnology.

### Serum biochemical parameters analysis

Blood samples were placed for 12 h at 4°C. After centrifugation (3000 g, 4°C, 15 min), the serum was collected for enzyme activity detection, and the remaining serum was stored at −80°C. Serum iron was assayed by colorimetric method. Lysozyme was measured by turbidimetric method. Superoxide gasification enzyme was measured by the hydroxylamine method. Test kits were purchased in Nanjing Jiancheng Bioengineering Institute. The determination was carried out according to the manufacturer’s instructions. The experiment was performed in triplicates.

### RNA extraction and qRT-PCR analysis

The expression levels of iron-related genes *hepcidin* and *ferroportin*, and the expression levels of immune-related genes *IL-1β, IL-10, TNF-α* and *MHC-II* were detected in hepatopancreas, spleen and intestine. Total RNA was extracted from with RNAiso Plus kit (Takara, Dalian, China) according to the manufacturer’s instructions, followed by DNase I treatment. Total RNA was quantified by absorbance ratio of 260 nm and 280 nm with NanoDrop 2000 spectrophotometer (Thermo Scientific, Waltham, USA), and the quality was evaluated using 1% agarose gel electrophoresis. The cDNA was synthesized using the HiScript^®^ II Q Select RT SuperMix (Vazyme, Nanjing, China) according to the manufacturer’s instruction for subsequent qPCR. All the cDNA products were diluted to 250 ng/µL, and the qPCR was performed using the AceQ^®^ qPCR SYBR^®^ Green Master Mix (Vazyme, Nanjing, China) on a Roche LightCycle^®^ 480 System (Roche, Basel, Switzerland) according to the manufacturer’s instructions. The primers mentioned in previous literatures are shown in **Table 2**. A melting curve was constructed for every qPCR product to confirm the specificity of the assays. A dilution series were prepared to check the efficiency of the reaction. 18S rRNA and β-actin were used as the housekeeping genes, and the relative mRNA expression level was calculated with the 2^-ΔΔCT^ method (34).

**Table 2:** Primers used in this study.

Gene	Primer	Primer Sequence (5′-3′)
*Hepcidin*	Forward	CAGCCGTTCCGTTCGTACA
	Reverse	AGCCTTTGTTACGACAGCAG
*Ferritin*	Forward	TCCTGTGCTTCGTGCGTGT
	Reverse	ACCTTCAGTCCGTCCTCGTG
*Ferroportin*	Forward	CCTCGGACATGCTCTGTCAA
	Reverse	CAGTCCATACACGGCTGTCA
*IL-1β*	Forward	AAGTTCCCGCTTTGGAGAGTA
	Reverse	GCCACATACCAGTCGTTCAGT
*IL-10*	Forward	CTCAAGCGGGATATGGTCAAA
	Reverse	TGGCAGAATGGTCTCCAAGTAG
*MHC-II*	Forward	ACAAGCCTCAGTGTGACGAG
	Reverse	TGTGTCCGGAATCTCATGGC
*TNF-α*	Forward	ATTTATCTCGGTGCGGCCTT
	Reverse	GCTTACAGAGCAAACACCCC
*18S rRNA*	Forward	ATTTCCGACACGGAGAGG
	Reverse	CATGGGTTTAGGATACGCTC
*β-actin*	Forward	GATGATGAAATTGCCGCACTG
	Reverse	ACCGACCATGACGCCCTGATGT

### DNA extraction and 16s rRNA gene sequencing

Microbial genomic DNA was extracted from intestinal contents by using PowerFecal^®^ DNA Isolation Kit (Mo Bio, CA, USA) following manufacturer’s instruction. DNA concentrations and purity were assessed with a NanoDrop 2000 UV-vis spectrophotometer (Thermo Scientific, Wilmington, USA). DNA quality was checked by 1% agarose gel electrophoresis. The V3-V4 hypervariable regions of the bacteria 16S rRNA gene were amplified with primers 341F (5’-CCTAYGGGRBGCASCAG-3’) and 806R (5’-GGACTACHVGGGTWTCTAAT-3’) by thermocycler PCR system (GeneAmp 9700, ABI, USA). The PCR conditions were as follows: 3 min of denaturation at 95 oC, 27 cycles of 30 s at 95°C, 30 s for annealing at 55°C, and 45s for elongation at 72 oC, and a final extension at 72°C for 10 min. PCR was performed in triplicate in a total volume of 20 µL containing: 4 µL of 5 × FastPfu Buffer, 2 µL of 2.5 mM dNTPs, 0.8 L of each primer (5 µM), 0.4 µL of FastPfu Polymerase and 10 ng of template DNA. Finally, add ddH2O to 20 μL. PCR products were gel purified using a 2% agarose gel and the AxyPrep DNA Gel Extraction Kit (Axygen Biosciences, Union City, CA, USA) and quantified using QuantiFluor ™ -ST (Promega, USA) according to the manufacturer’s protocol. Purified amplicons were pooled in equimolar and paired-end sequenced on an Illumina MiSeq PE300 platform (Illumina, San Diego, USA), according to the standard protocols by Majorbio Bio-Pharm Technology Co. Ltd. (Shanghai, China).

Raw FASTQ files were de-multiplexed using in-house perl script, and then quality-filtered by fastp version 0.19.6 and merged by FLASH version 1.2.7 with the following criteria: (i) the 300 bp reads were truncated at any site receiving an average quality score of<20 over a 50 bp sliding window, and the truncated reads shorter than 50 bp were discarded, reads containing ambiguous characters were also discarded; (ii) only overlapping sequences longer than 10 bp were assembled according to their overlapped sequence. The maximum mismatch ratio of overlap region is 0.2. Reads that could not be assembled were discarded; (iii) reads containing ambiguous bases were removed; and (iv) sequences with >= 10 bp overlap longer were merged. Operational taxonomic units (OTUs) were clustered with 97% similarity cut-off using UPARSE 7.1 and chimeric sequences were identified and removed using UCHIME. Taxonomy was assigned by RDP classifier algorithm against the Silva 16S rRNA database using a confidence threshold of 70%. The average read length was approximately 400 bp after quality trimming. A phylogenetic tree was constructed by the neighbor joining method with CLUSTAL X and MEGA 6.0.

Bioinformatic analyses of gut microbiota was performed on the Majorbio Cloud Platform (https://cloud.majorbio.com). Based on the OTUs information, rarefaction curves and alpha diversity indices including observed OTUs, Chao1 richness, and Shannon index were calculated with Mothur v1.30.1. The similarity among the microbial communities in different samples was determined by principal coordinate analysis (PCoA) based on Bray-curtis dissimilarity using Vegan v2.5-3 package. All 16S rRNA sequence data used in this study were deposited in the Sequence Read Archive (SRA) of the National Center for Biotechnology Information (NCBI) under the BioProject accession number PRJNA900049.

### Statistical analyses

The results were expressed as the means ± standard deviation. We analyzed the experimental data through Multi-factorial ANOVA using the SPSS software (*p*<0.05). Different superscript letters in each group (a, b, and c) denote significant variations. The mortality rate was analyzed by Mantel-Cox test. Use the following formula to calculate: Weight gain (WG), specific growth rate (SGR), condition factor (CF), hepatosomatic index (HSI), visceral body ratio (VIS) and the mortality rate were calculated by the following formula:


$$\text{WG}(\%)=100\times ({\text{W}}_{2}-{\text{W}}_{1})/{\text{W}}_{1}$$

$$\text{SGR}(\%)=100\times ({\text{lnW}}_{2}-{\text{lnW}}_{1})/{\text{TW}}_{1}$$

$$\text{CF}(\%)=100\times \text{body weight}(\text{g})/\text{body length}{\left(\text{cm}\right)}^{3}$$

HSI(%)=100×hepatopancreas weight(g)/body weight(g)

VIS(%)=100×Visceral weight(g)/body weight(g)

$$\text{Cumulative mortality}(\%)=100\times ({\text{N}}_{0}-{\text{N}}_{\text{F}})/{\text{N}}_{0}$$

where W_1_ is the initial weight (g), W_2_ is the final weight (g), T is time (d), N_0_ is the initial number of fish and N_F_ is the final number of fish.

## Results

### Stability of Cihep proteins

**Figure 1:**
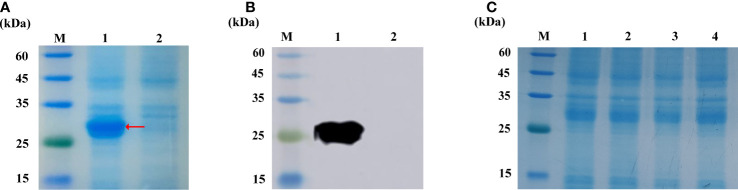
Expression and activity verification of Cihep. **(A)** Analysis by SDS-PAGE. M=protein marker; 1= pGEX-KG-hepcidin after overnight induction; 2= pGEX-KG-hepcidin before induction. **(B)** Western blotting assay of anti-GST antibody. M=protein marker; 1= pGEX-KG-hepcidin after overnight induction; 2= pGEX-KG-hepcidin samples before induction. **(C)** Protein lyophilized powder stability at -20°C. M=protein marker; 1, 2, 3, 4= represent D1, D7, D14 and D28 respectively.

We obtain the Cihep fusion protein in the prokaryotic expression system. A target band of 28 kDa was confirmed by SDS-PAGE (**Figure 1A**) and Western blot (**Figure 1B**). To determine the stability of Cihep protein, the freeze-dried powder was stored at -20°C refrigerator and sampled at day 1, 7, 14 and 28, respectively. Then, the stability of Cihep protein was determined by SDS-PAGE (**Figure 1C**). The results showed that the properties of the protein remained unchanged at -20°C for 28 days.

### Synergistic effect of Cihep and CS

**Figure 2:**
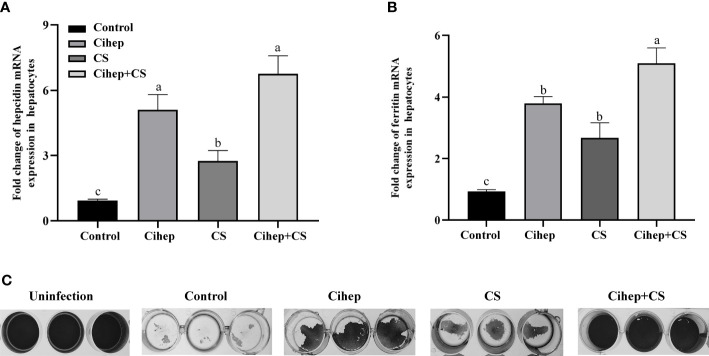
Preventive effects of Cihep and CS in L8824-infected bacteria by iron metabolism. **(A)** qRT-PCR assay of mRNA level of *hepcidin* in hepatocytes treated with Cihep and CS. **(B)** Fluorescence assay of LIP level in hepatocytes treated with Cihep and CS. **(C)** Bacterial suspension in M199 was incubated with L8824 cells for 3 h and then treated with PBS, Cihep, CS and Cihep+CS at 28°C for 24 h. Cell density was determined using crystal violet staining. Uninfected cells were used as blank control. Different small letters in each group (a, b, and c) denote significant variations suggested by the multifactorial ANOVA statistics (p < 0.05).

The results showed that hepcidin mRNA was significantly increased after incubation of chitosan with hepatocytes (**Figure 2A**). Subsequently, we examined the change in the labile iron pool (LIP) after 12 h Cihep and CS incubated hepatocytes (**Figure 2B**). The results showed that Cihep and CS stimulated the cells to increase the intracellular LIP content. To further investigate whether hepcidin and chitosan have synergistic effects, we investigated the preventive effect of hepcidin and chitosan on L8824 cells infected with *F. columnare*. Crystalline violet staining showed that large amount of cells died in the control group, while the Cihep+CS group had less cells death (**Figure 2C**). After 3 h of infection with *F. columnare*, all the hepcidin treatment group, chitosan treatment group, and hepcidin combined with chitosan treatment group had obvious protective effect, among which, the hepcidin combined with chitosan treatment group had the most significant protective effect, indicating that hepcidin and chitosan had a good synergistic effect.

### Growth performance

The growth performance parameters of grass carp fed with supplemented with hepcidin were summarized in **Table 3**. Compared with those in the control group, the WG, SGR, CF, and HSI in four experience groups were increased. Among all five groups, Cihep-3d group exhibited the highest WG, SGR, and VIS, and the optimal weight gain effect.

**Table 3:** Effects of dietary supplementation of Cihep and CS on growth performance of grass carp.

Items	Control	Cihep-3d	Cihep-7d	Cihep+CS-3d	Cihep+CS-7d
Initial weight (g)	150.50 ± 1.80	149.53 ± 0.60	149.71 ± 1.30	149.15 ± 0.70	149.33 ± 0.60
Final Weight (g)	401.82 ± 7.35^b^	408.56 ± 3.90^a^	403.75 ± 8.52^b^	402.15 ± 11.91^b^	400.83 ± 5.20^b^
Weight gain (WG, g)	166.97 ± 4.60^b^	173.27 ± 2.70^a^	169.62 ± 3.90^b^	169.67 ± 8.90^b^	168.51 ± 2.50^b^
Specific growth rate (SGR, %/d)	1.71 ± 0.03^b^	1.79 ± 0.02^a^	1.77 ± 0.03^b^	1.77 ± 0.06^b^	1.76 ± 0.02^b^
Condition factor (CF, g/cm^3^)	1.13 ± 0.10^b^	1.23 ± 0.11^a^	1.15 ± 0.20^b^	1.16 ± 0.10^b^	1.15 ± 0.12^b^
Hepatosomatic index (HSI, %)	1.65 ± 0.24^c^	1.70 ± 0.42^b^	1.78 ± 0.32^a^	1.71 ± 0.30^b^	1.70 ± 0.30^b^
Visceral body ratio (VIS, %)	8.85 ± 0.60^b^	9.09 ± 0.82^a^	8.86 ± 1.02^b^	8.85 ± 0.70^b^	8.57 ± 0.80^b^

Value are presented as mean ± SD. Means in the same row with different superscripts are significantly different (P< 0.05).

### Reduction in mortality and establishment of a protective immune state by Cihep and CS in grass carp

**Figure 3:**
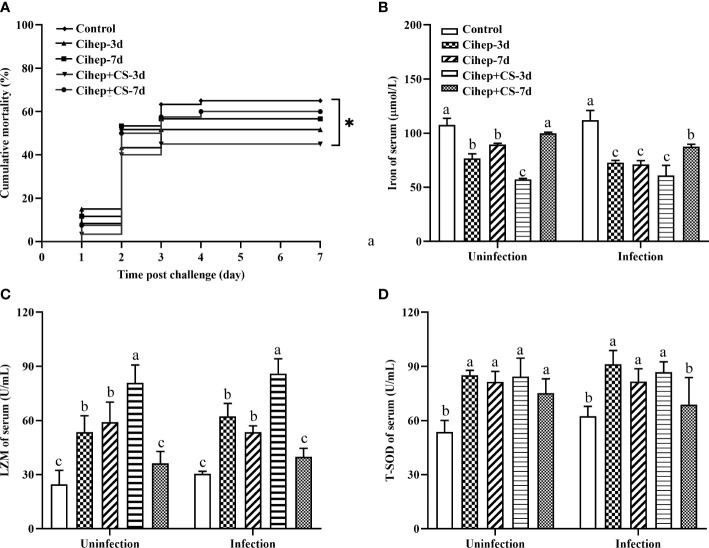
Mortality and Serum innate immune indexes statistics. **(A)** Fish in each group (n = 20) were challenged with 500 µL F. columnare (1 × 10^9^ CFU/mL), and death events in each group were monitored on the next 7 days. p values were calculated by Log-rank (Mantel–Cox) Test (* p< 0.05). **(B-D)** Serum innate immune indicators. Iron of serum **(B)**, LZM **(C)** and T-SOD **(D)** were measured by commercial kits (Nanjing Jiancheng Institute of Biological Engineering, Nanjing, China). Different small letters in each group (a, b, and c) denote significant variations suggested by the multifactorial ANOVA statistics (p < 0.05).

After bacterial challenge, the mortality rate of the four experimental groups was lower than that of the control group (65.00%). Among them, Cihep+CS-3d group displayed the lowest mortality rate (45.00%) (**Figure 3A**). Results showed the mortality rate in the Cihep+CS-3d group was significantly lower than that of the control group (*p*<0.05). Our results shows that hepcidin and CS have a significant preventive effect.

Before and after challenge, the serum iron in all four experimental groups was lower than that in the control group. The serum iron level was extremely significantly lower in the Cihep + CS-3d group than in the control group (**Figure 3B**). The trend of changes in LZM (**Figure 3C**) was significantly higher. The serum enzyme activity of Cihep+CS-3d group was significantly higher than those of the other four groups before and after challenge (**Figure 3C**). The T-SOD activity of Cihep-3d group and Cihep + CS-3d group was significantly higher than that of the control group (**Figure 3D**). Overall, the Cihep-3d and Cihep+CS-3d groups, especially the Cihep+CS-3d group, can effectively exhibit higher serum innate immunity levels than other groups.

### Protective effect of Cihep and CS against pathological damage of gill tissue

**Figure 4:**
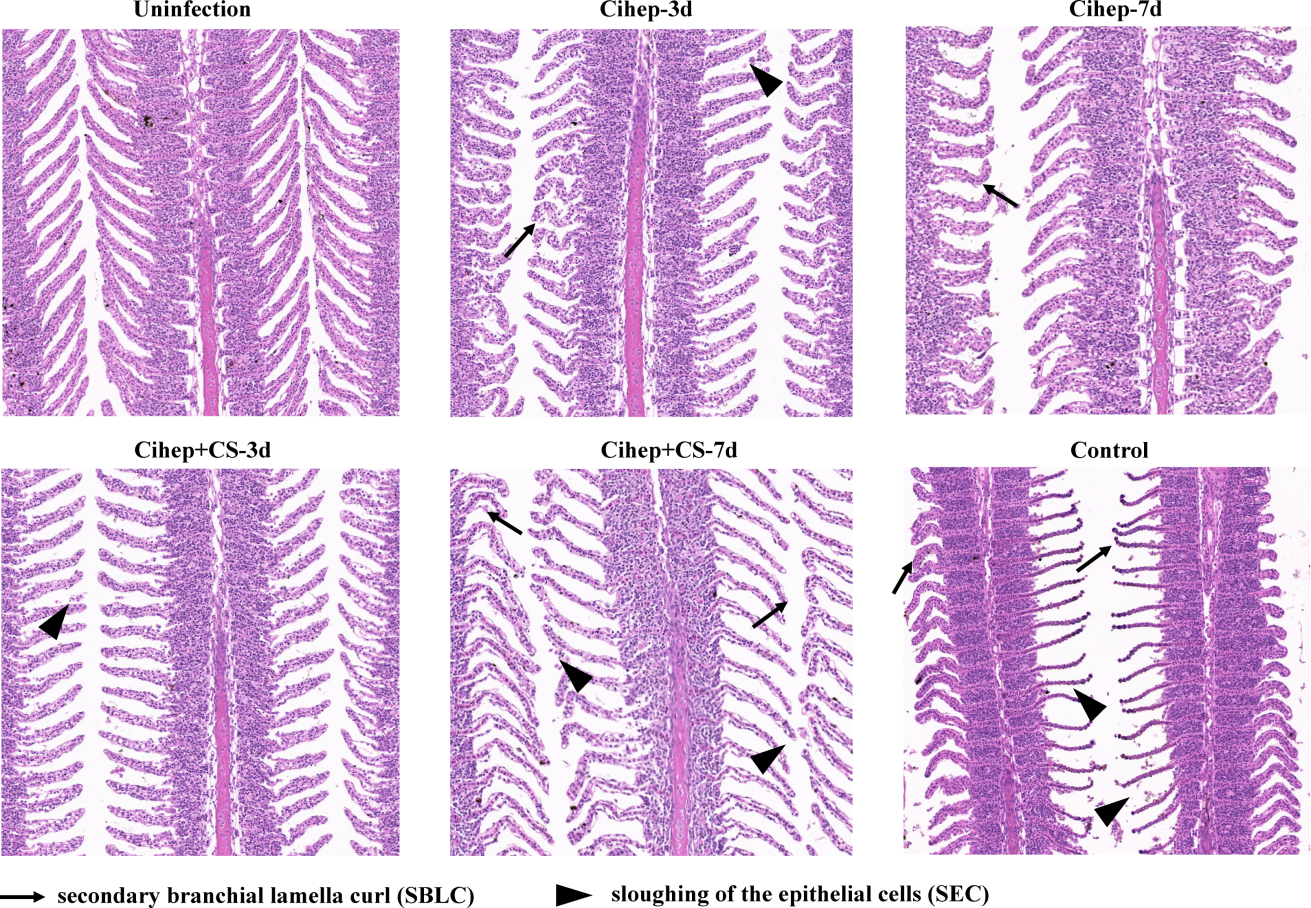
Gill tissue lesion post bacterial infection in grass carp. HE staining of gill, bar = 120 μm. Secondary branchial lamella curl (
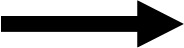
), Sloughing of the epithelial cells (
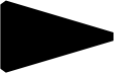
), Epithelial hyperplasia (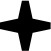).

The healthy gill filaments were neatly distributed and the gill lamellae were well arranged. At 7 d post infection, the gill tissue epithelial cells of the control group were severely detached, and necrotic. The gill filaments showed symptoms such as bleeding and curling. Compared with the control group, the lesions in the four experimental groups were mild and the gill lamellae were more neatly arranged (**Figure 4**).

### Alleviation of *F. columnare*-induced inflammation by Cihep and CS

The expressions of *IL-1β*, *TNF-α* and *MHC-II* in the hepatopancreas were significantly higher in the four experimental groups than in the control group after injection with *F. columnare* (**Figure S1A**). In the spleen, the expression level of *IL-1β* was also significantly higher in four experimental groups than that the control group. In addition, the expression level of *IL-10* in the spleen was significantly lower than that in the control group (**Figure S1B**). In the intestine, the expression levels of *IL-1β*, *TNF-α* and *MHC-II* were also significantly higher in four experimental groups than in the control group.

### Regulation of individual iron levels by Cihep and CS

**Figure 5:**
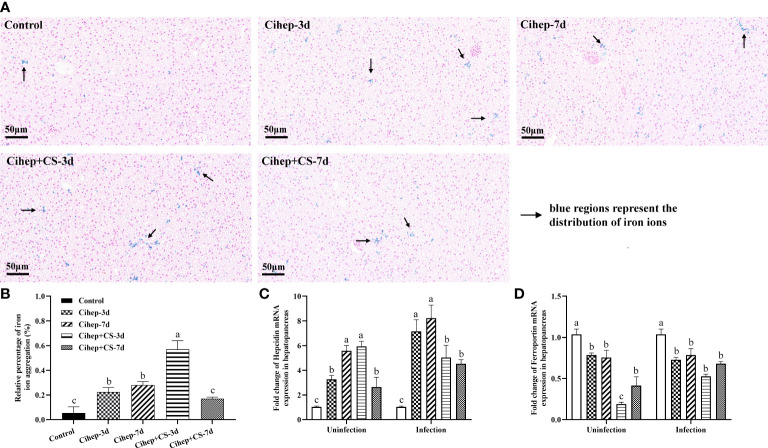
Regulation of hepatopancreas iron and iron metabolism-related genes by Cihep and CS. **(A)** Iron distribution in hepatopancreas. Using the PBS group as the control, the iron content in hepatopancreas was stained by Prussian blue, bar = 50 μm. Arrowheads in the image show the iron content in hepatopancreas. **(B)** The Prussian blue-stained sections from **(A)** were analyzed with ImageJ image-analysis software to calculate the relative percentage of iron ion aggregation. **(C)** mRNA expression expression of hepcidin gene in hepatopancreas. **(D)** mRNA expression expression of ferroportin gene in hepatopancreas. Different small letters in each group (a, b, and c) denote significant variations suggested by the multifactorial ANOVA statistics (p < 0.05).

The obvious blue was observed in the four experimental groups, but much less obvious in the control group, indicating that adding hepcidin to the diet increased the accumulation of iron ions in the hepatopancreas of grass carp (**Figure 5A**). The results showed that iron ion aggregation was significantly higher in the Cihep+CS-3d group than in the other three groups (*p*<0.01).

Before challenge, in the hepatopancreas, the expression level of *hepcidin* was significantly higher in both Cihep -7d and Cihep + CS-3d groups than in the other groups (**Figure 5C**), but the expression level of *ferroportin* was lower than that in the control group (**Figure 5D**). After challenge, the expression levels of *hepcidin* were significantly higher in the four experimental groups than in the control group (**Figure 5C**).

### Improving the diversity gut microbial by Cihep and CS in grass carp

**Figure 6:**
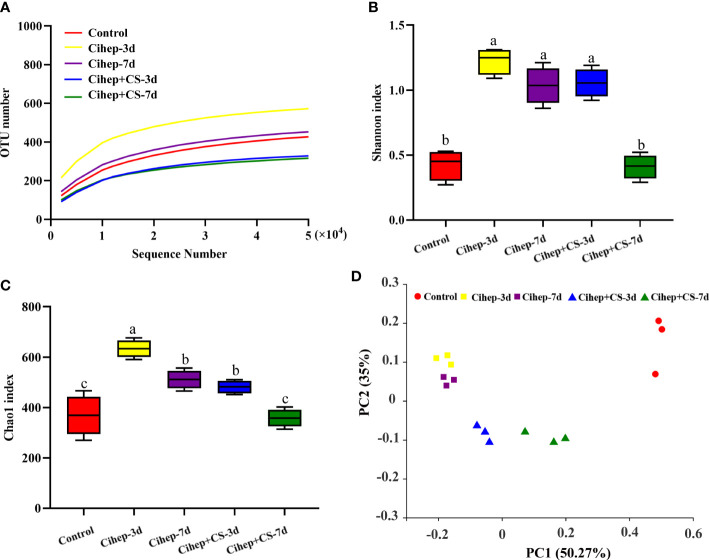
Intestinal 16S sequencing analysis of grass carp. **(A)** Rarefaction Observed species curves of OTUs clustered at 97% sequence (Y-axis indicates number of observed species). **(B, C)** Alpha-diversity index difference based on Chao1 metric and. Shannon index. **(D)** Beta-diversity index difference based on unweighted UniFrac. Different small letters in each group (a, b, and c) denote significant variations suggested by the multifactorial ANOVA statistics (p < 0.05).

As shown in **Figure 6A**, the stable curves indicated that the sequencing results were reliable. The alpha diversity of samples was analyzed based on Chao 1 index and Shannon index. As shown in **Figures 6B, C**, the Shannon index and Chao 1 index in Cihep-3d group and Cihep+cs-3d groups were significantly higher than those in the control group, but there was no difference between Cihep-3d group and Cihep+CS-3d group. As shown in **Figure 6D**, all samples of Cihep-3d group and Cihep-7d group were clustered together, and the samples of Cihep + CS-3d group and Cihep + CS-7d group were divided into two different clusters. Moreover, the samples of the four experimental groups and the control group are divided into different clusters, indicating that there are significant differences in gut microbiota between the experimental groups and the control group. In addition, we also found that the gut microbiota communities of grass carp fed with hepcidin alone and hepcidin + chitosan were significantly separated.

**Figure 7:**
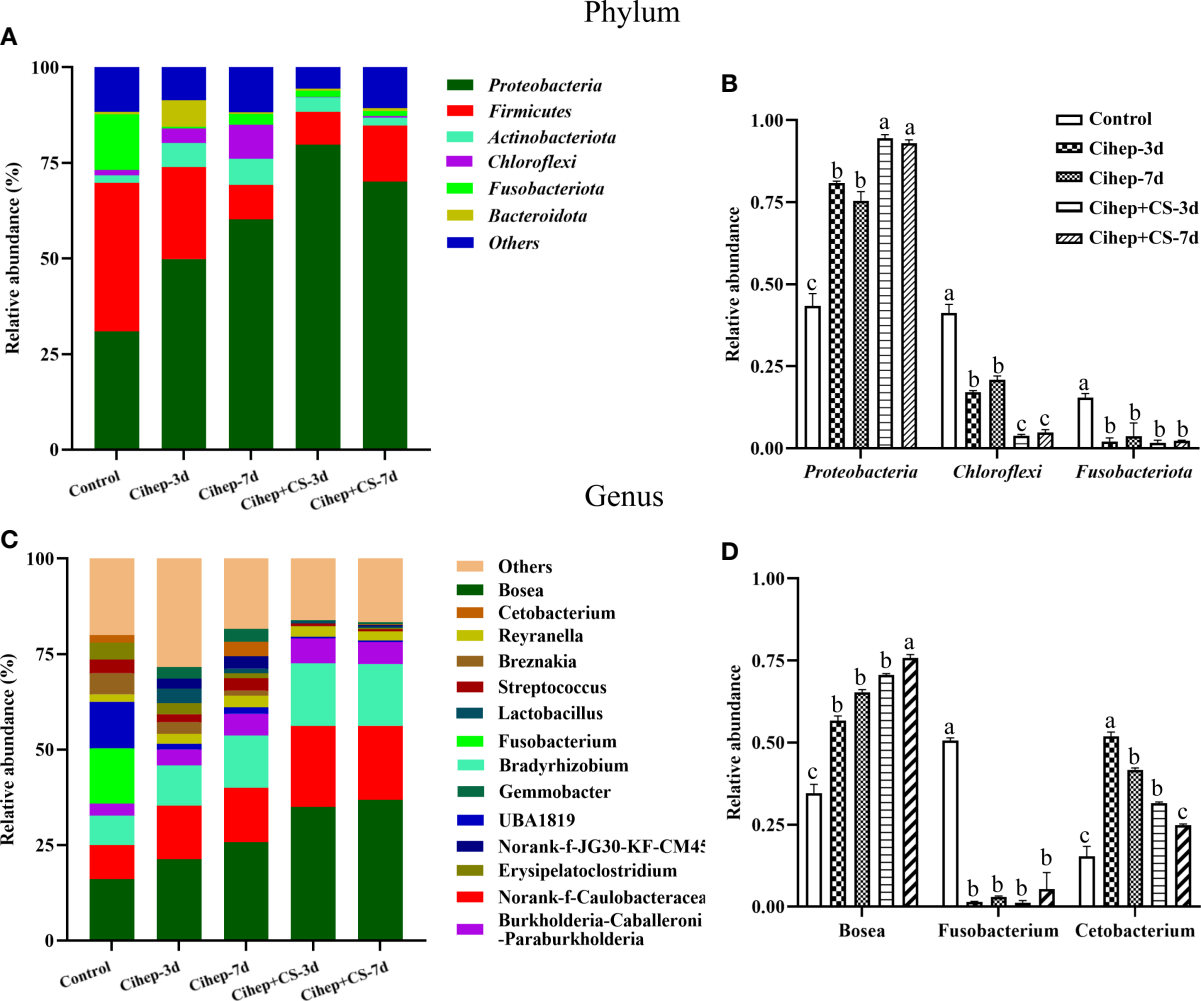
Sequencing analysis of gut microbiota species relative abundance in grass carp. **(A, B)** Relative abundance in phylum level. “Others” includes the sum of different phyla which are less than 1% in the sample. The representative differential phyla were statistically analyzed. **(C, D)** Relative abundance in genus level. “Others” includes the sum of different genera which are less than 1% in the sample. The representative differential genera were statistically analyzed. Different small letters in each group (a, b, and c) denote significant variations suggested by the multifactorial ANOVA statistics (p < 0.05).

**Figure 8:**
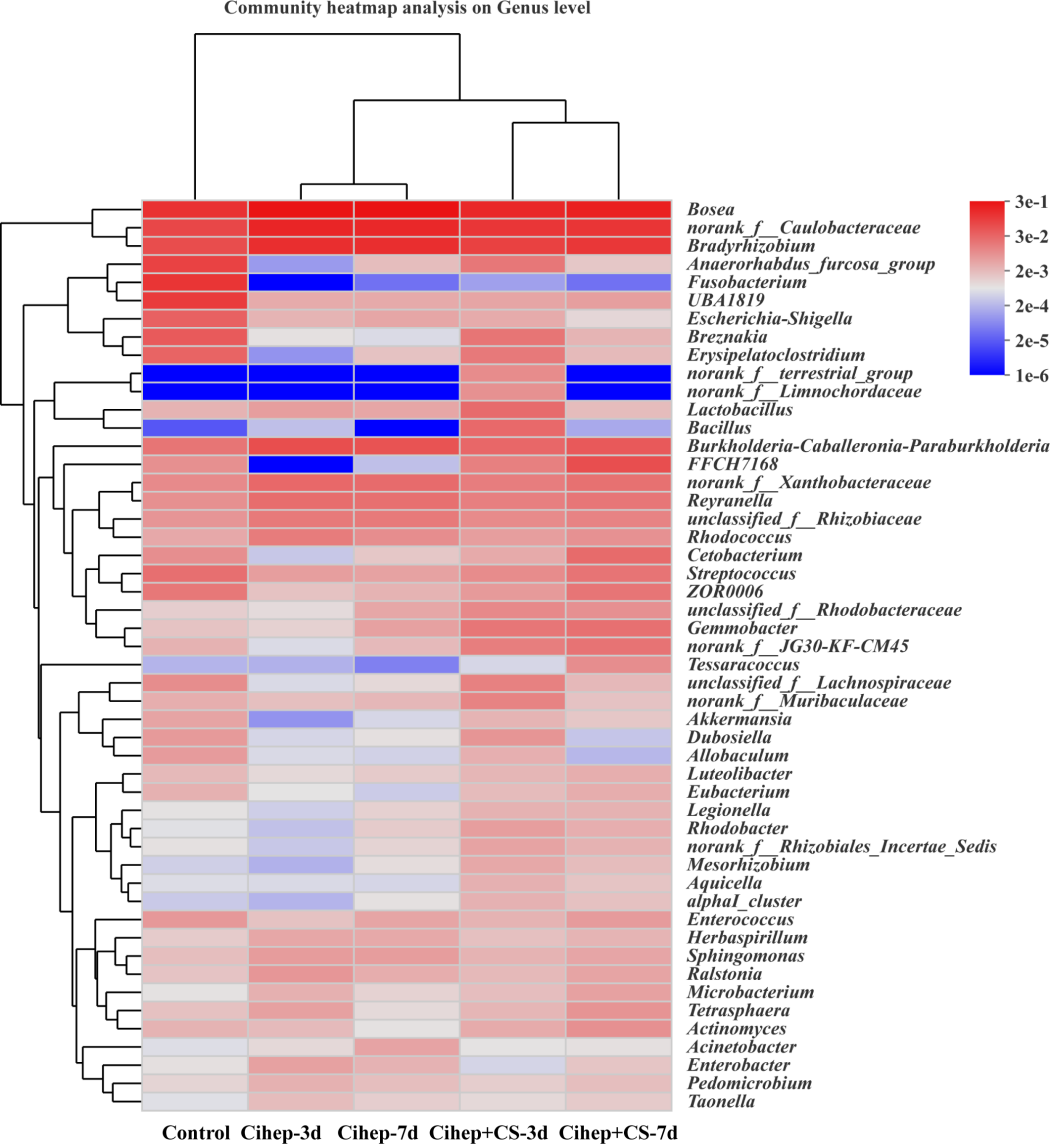
Heatmap of hierarchy cluster results for the relative abundance of all samples at genus level. The sample information is shown along the horizontal axis. Annotations are shown along the vertical axis on the right. The genera clustering tree is shown along the vertical axis on the left. The sample clustering tree is shown above. The color represents the Z-Score, which is the standardized relative abundance.

The distribution of gut microbiota of grass carp in each group was shown in **Figure 7**. At the phylum level (**Figure 7A**), the gut microbiota of grass carp mainly included *Proteobacteria*, *Firmicutes*, *Actinobacteria*, and *Chloroflexi*. Among them, the abundance of *Proteobacteria* in the four experimental groups was significantly higher than that in the control group, but the abundance of *Fusobacteria* and *Chloroflexi* was significantly lower (**Figure 7B**). At the genus level (**Figure 7C**), Bosea and Caulobacteraceae-unclassified exhibited the highest abundance in the five groups. The abundance of Bradyrhizobium in the four experimental groups was significantly higher than that in the control group. It should be noted that Fusobacterium only existed in the control group (**Figure 7D**). Hierarchical cluster heatmap analysis showed that the gut microbiota in the control group and the experimental groups were not clustered in the same branch (**Figure 8**). In the Cihep + CS-3d group, the content of beneficial bacteria such as Lactobacillus and Bacillus increased significantly, while the content of harmful bacteria such as Enterobacter decreased significantly.

## Discussion

Hepcidin is an antimicrobial peptide expressed in the liver. It has an ability to inhibit a variety of pathogens, regulate gut microbiota, and enhance immune functions (35–37). Many studies have revealed that antimicrobial peptides as feed additives can have a positive effect on the growth performance of numerous farmed animals (38–40). In this experiment, we found that the addition of hepcidin to the feed improved the growth performance and immunity function of grass carp. The highest WG, and SGR were observed in Cihep-3d group and Cihep+CS-3d group, and the second highest WG in the Cihep-7d group and Cihep+CS-7d group. This is in line with the previous findings that antimicrobial peptides can significantly improve growth performance in cultured fish (41–43).

Bony fish are at the lower stage of phylogeny and the acquired immune system is not developed enough. The innate immune system plays a crucial role in the resistance of fish to invasion by pathogenic organisms. The mortality is an important indicator reflecting organism’s resistance to disease. The mortality of grass carp in the control group was higher than that in the four experimental groups after infection with *F. columnare*, indicating that the addition of hepcidin into feed can enhance the resistance of grass carp to *F. columnare* infection. Our results are in line with the previous report that the addition of Tilapia hepcidin 2-3 to grouper feed activated the innate immune system and significantly increased the survival rate of grouper infected with *V. alginolyticus* (44). The health status of the gills is highly dependent on the antioxidant capacity and immunity of the fish body, which can be well reflected by the disease resistance (45, 46). In this experiment, the disease resistance could be widely determined by intraperitoneal injection of *Flavobacterium columnare*. The mortality of grass carp after challenge was significantly reduced and the pathological symptoms of gill tissue were significantly improved by feeding hepcidin and chitosan. In addition, the activity of superoxide dismutase and the expression of immune genes were also significantly increased. These results suggested that Cihep and CS could significantly enhance serum enzyme activity and immune gene regulation compared with the control group.

Hepcidin, a major regulator of systemic iron homeostasis, regulates the expression of ferroportin on cell surface, thus reducing dietary iron uptake, eventually lowering plasma iron concentrations (17). The hepatopancreas is the main tissue synthesizing ferritin and storing iron (47). All biological processes require the involvement of iron, and iron ions play a key role in the interaction between the host and the pathogen (48, 49). Based on this, we hypothesized that iron regulators could prevent fish diseases by regulating iron metabolism.

Gut microbiota is a key factor in body development, immunity, and nutrient transformation (50). Research has proven that microbial-directed therapy is an excellent approach to improve the health of the host (51). Diet is the main factor affecting the proportion and content of microbiota in the gut (52). The gut microbiota of fish are remarkably plastic and can enhance host health by applying exogenous substances to improve the existing microbiota of fish (53). Therefore, this study focused on the correlation between gut microbiota and fish health. We found that hepcidin increased the gut microbiota α-diversity and β-diversity, thus significantly altering the community composition of grass carp gut microbiota. The microbial community with the highest abundance in the gut of grass carp mainly included *Proteobacteria*, *Firmicutes*, *Actinobacteria*, and *Chloroflexi* in our study. Further, we found that the abundance of *Firmicutes* and *Bacteroidota* was significantly higher in the Cihep + CS-3d group than in the other groups. Short-chain fatty acids (SCFAs) are reported to be produced mainly by *Firmicutes* and *Bacteroidetes*, and SCFAs have anti-inflammatory effects to prevent metabolic diseases, especially acetate, propionate and butyrate which promote the absorption of inorganic ions in the intestine and have a role in the gut microbiota (54). Dietary supplementation of sodium butyrate has been reported to improve growth performance, antioxidative capacity, and intestinal absorption in grass carp (55). Butyrate supplementation to snapper feed also has a promotion effect on growth performance and intestinal metabolism (56). The genus Cetobacterium is one of the main bacterial species in the gut of herbivorous fish, and it is related to the digestive function of fish (57), which might explain why our Cihep-3d group achieved the most weight gain (WG) in grass carp.

The 3-day consecutive feeding followed by 3-day interval exhibited better protection effect than the 7-day consecutive feeding followed by 7-day interval. Immunological indicators of serum reflect the level of immunity of the body (58). In this experiment, compared with the control group, the activity of lysozyme in the serum of grass carp was higher at before and after infection. In particular, the up-regulation of lysozyme activity in Cihep -3d group and Cihep+CS-3d group was the most obvious after infection. Lysozyme, as a strong antibacterial enzyme, can inhibit the proliferation and colonization of bacteria by disrupting cell wall polysaccharides, thus leading to bacterial damage and death, and Lysozyme participates in body defense and immune regulation, thus it is an important indicator of fish innate immunity (59–61). In this study, the T-SOD content in Cihep-3d group and Cihep+CS-3d group was higher than that in the other three groups before and after the challenge. When bacterial infection in fish body causes inflammation, the body produces the large number of free radicals to resist the invasion of pathogenic bacteria (62). The accumulation of free radicals will cause the lipid peroxidation of the cell membrane, thus leading in membrane fission, eventually causing cell damage and even death (63). T-SOD is the most important and the best free radical scavenger in the organism, and it can maintain the metabolic balance of the body (7, 64). The above findings jointly explain why 3-day consecutive feeding followed by 3-day interval significantly improved the viability of grass carp. Therefore, our feeding mode is a very meaningful feeding strategy, which can provide an important reference for adding immune enhancers in aquaculture.

Our data indicated that the combined feeding of hepcidin and chitosan exhibited a significant better protective effect than feeding of hepcidin alone. Chitosan is a common feed additive due to low toxicity, high biocompatibility, low cost, and easy-to-handle properties (65, 66). The addition of chitosan to the diet of juvenile crucian carp (*Carassius auratus*) has significantly reduced the abundance and diversity of intestinal bacteria (67). Similarly, the addition of chitosan to the diet has a regulatory effect on certain non-specific immune functions (68, 69). These previous findings were supported by our results that the Cihep+CS-3d group had the lowest cumulative mortality. Chitosan can exert the immunostimulatory effect possibly by promoting the function of inflammatory cells and inducing an inflammatory response (70). We also found that the expression of *IL-1β* and *TNF-α* in the Cihep+CS-3d group was significantly higher than that in the control group. *IL-1β* and *TNF-α* are the most effective molecules in the innate immune system, and they jointly mediate the immune response (71, 72), which might explain why the combined application of hepcidin and chitosan can significantly improve the immunity of fish in our study. In conclusion, the protective effect of hepcidin in combination with chitosan is superior to that of hepcidin alone. Our findings provide a new perspective for the combined application of immune enhancers.

## Conclusion

In summary, this study showed that oral administration of recombinant hepcidin improved the growth performance and regulated the iron metabolism. The immunity and survival rate of grass carp were improved. The 3-day consecutive feeding followed by 3-day interval exhibited better protection effect than the 7-day consecutive feeding followed by 7-day interval, and hepcidin in combination with chitosan was better than that of hepcidin alone.

## Data availability statement

The datasets presented in this study can be found in online repositories. The names of the repository/repositories and accession number(s) can be found below: BioProject *via* accession ID: PRJNA900049.

## Ethics statement

The animal study was reviewed and approved by the Ethical Committee on Animal Research at Huazhong Agricultural University (ID Number: HZAUFI-2021-0012).

## Author contributions

JZ and MF conceived and designed the experiments, and wrote the manuscript. MF and WZ performed the experiments and analyzed the data. QZ and RK provided guidance on the experimental design. JS and GY revised the manuscript critically. All authors reviewed the manuscript. All authors contributed to the article and approved the submitted version.
