# Supplementary material for: Oral administration of hepcidin and chitosan benefits growth, immunity, and gut microbiota in grass carp (Ctenopharyngodon idella)
Source: Front Immunol. 2022 Dec 15;13:1075128. doi: 10.3389/fimmu.2022.1075128 (PMC9798086; doi:10.3389/fimmu.2022.1075128)
Supplement: Supplementary file 1 [file DataSheet_1.docx]

# Supplementary material


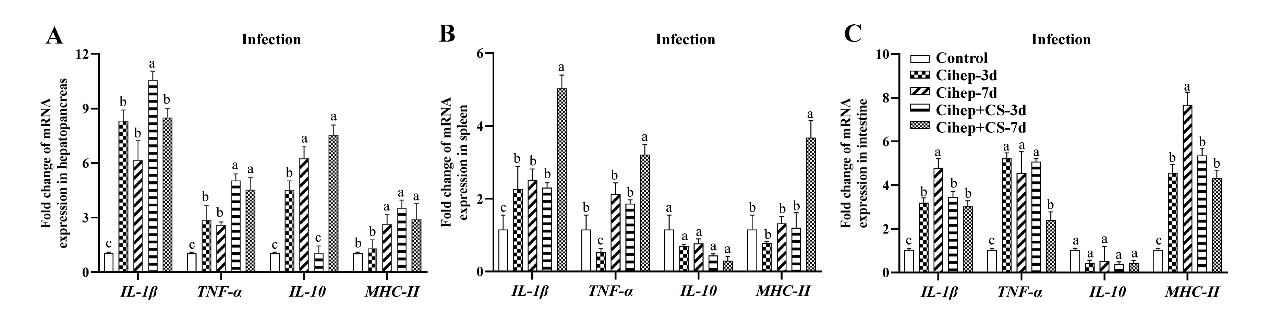


# Fig. S1. Relative expression of immune regulation genes in hepatopancreas (A), spleen (B), and intestine (C). The *IL-1β*, *TNF-α*, *IL-10* and *MHC-II* transcripts were checked by qRT-PCR. The 18S rRNA gene and β-actin were used as internal control genes.
